# Clinical, epidemiological, and spatial features of human rabies cases in Metro Manila, the Philippines from 2006 to 2015

**DOI:** 10.1371/journal.pntd.0010595

**Published:** 2022-07-19

**Authors:** Ferdinand D. Guzman, Yuta Iwamoto, Nobuo Saito, Eumelia P. Salva, Efren M. Dimaano, Akira Nishizono, Motoi Suzuki, Oladeji Oloko, Koya Ariyoshi, Chris Smith, Christopher M. Parry, Rontgene M. Solante

**Affiliations:** 1 San Lazaro Hospital, Manila, Philippines; 2 School of Tropical Medicine and Global Health, Nagasaki University, Nagasaki, Japan; 3 Department of Clinical Medicine, Institute of Tropical Medicine, Nagasaki University, Nagasaki, Japan; 4 Department of Microbiology, Faculty of Medicine, Oita University, Yufu, Japan; 5 Infectious Disease Surveillance Center, National Institute of Infectious Diseases, Tokyo, Japan; 6 Department of Clinical Research, London School of Hygiene and Tropical Medicine, London, United Kingdom; 7 Clinical Sciences, Liverpool School of Tropical Medicine, Liverpool, United Kingdom; Universitetet i Oslo, NORWAY

## Abstract

Rabies remains a public health problem in the Philippines despite the widespread provision of rabies vaccines and rabies immunoglobulin (RIG) as post-exposure prophylaxis (PEP). Detailed descriptions of recent human rabies cases in the Philippines are scarce. This study aimed to describe the clinical, epidemiological, and spatial features of human rabies cases between January 1, 2006, and December 31, 2015. We conducted a retrospective hospital-based case record review of all patients admitted to one referral hospital in Manila who received a clinical diagnosis of rabies. During the 10-year study period there were 575 patients (average 57.5 cases per year, range 57 to 119) with a final diagnosis of rabies. Most patients were male (n = 404, 70.3%) and aged ≥ 20 years (n = 433, 75.3%). Patients mostly came from the National Capital Region (n = 160, 28.0%) and the adjacent Regions III (n = 197, 34.4%) and IV-A (n = 168, 29.4%). Case mapping and heatmaps showed that human rabies cases were continuously observed in similar areas throughout the study period. Most patients had hydrophobia (n = 444, 95.5%) and/or aerophobia (n = 432, 93.3%). The leading causative animals were dogs (n = 421, 96.3%) and cats (n = 16, 3.7%). Among 437 patients with animal exposure history, only 42 (9.6%) had been administered at least one rabies vaccine. Two patients (0.5%), young children bitten on their face, had received and a full course of rabies vaccine. Human rabies patients were continuously admitted to the hospital, with no notable decline over the study period. The geographical area in which human rabies cases commonly occurred also did not change. Few patients received PEP and there were two suspected cases of PEP failure. The retrospective design of this study was a limitation; thus, prospective studies are required.

## Author summary

Rabies remains a public health problem in the Philippines despite improvements in the availability of rabies vaccines and rabies immunoglobulin (RIG) as post-exposure prophylaxis (PEP). The incidence of rabies is highest in Metro Manila and surrounding areas. We reviewed the records of all human rabies patients admitted to the national infectious disease hospital in Manila between 2006 and 2015. This hospital treats most cases in this area. During the 10-year study period, human rabies cases were continuously admitted to the hospital, with no notable decline in numbers by year. Most patients were adult men bitten by domestic dogs. The geographical areas in which cases commonly occurred during the 10-year period also did not change over time. Only 9.6% of patients had received at least one dose of a rabies vaccine as PEP. Although the risk of PEP failure is reported to be almost zero, we identified two suspected cases of PEP failure. The retrospective design of this study was a limitation, and the exact details of PEP were not reliably available. As human rabies death is a significant public health concern, the circumstances of each case should be prospectively investigated. Further research is required to understand how to reduce the number of rabies cases.

## Introduction

Rabies is a zoonotic viral infection of the central nervous system caused by members of the *Lyssavirus* genus, principally rabies virus, which results in fatal encephalomyelitis [1,2]. Rabies imposes a significant public health burden worldwide, particularly in developing countries where domestic dogs are the main reservoir for disease transmission to humans [3]. Over 55,000 rabies deaths are estimated per year, with 95% seen in Asia and Africa [4].

Following bite exposure from a rabid animal, human rabies is almost entirely preventable through the administration of proper post-exposure prophylaxis (PEP). PEP regimens are selected according to the World Health Organization (WHO) category of bite exposure [5]. The PEP required for Category III exposure includes wound care, a series of rabies vaccines and direct wound infiltration with rabies immunoglobulin (RIG) [5,6]. Since the introduction of a cost-effective multi-site intradermal (ID) vaccination, this regimen has been widely adopted in many low-to middle-income countries [7]. The Philippines was one of the earliest countries to introduce the ID regimen in 1997 [8,9]. The Department of Health in the Philippines initiated and expanded a decentralized network of animal bite treatment centers (ABTC) where patients can receive PEP, and the number of ABTC and patients receiving PEP has increased since 2005 [9–11]. The annual number of people receiving PEP and registered in the national system increased sharply from 176,501 in 2007 to 328,733 in 2011 and 783,663 in 2015 (S1 Fig) [10,11]. Despite intensive efforts to treat animal bite victims in the Philippines, 200–300 rabies deaths have been reported each year since 2007 [10,12]. Achieving the goal of the global strategic plan, namely “Zero by 30”, requires strengthening the control program based on scientific analysis [13].

San Lazaro Hospital (SLH), based in Manila, is a 500-bed hospital that serves as the national referral center in the Philippines for infectious diseases and tropical medicine. The hospital admits approximately 60–80 human rabies cases each year and is one of the main hospitals providing PEP in Manila [14]. According to 2018 national data, SLH treated 62% (n = 64) of human rabies patients in the National Capital Region (NCR), Region III, and Region IV-A [9–11]. In a previous study conducted at the SLH between January 1987 and June 2006, 1,839 patients with human rabies were admitted [14]. The study showed that only 31 (1.7%) patients received at least one rabies vaccine as PEP and none of the rabies patients had received a full PEP course [14]. While the availability of rabies vaccines and RIG has gradually increased since 2006, no study has described the impact of improvements in rabies care and PEP treatments on human cases. The main objective of this study was to describe the clinical and epidemiological features of patients with human rabies admitted to SLH since the study by Dimaano et al [14] and to compare the characteristics of patients with rabies between 2006 and 2015 to those admitted between 1987 and 2006. The second objective was to analyze the changing distribution of human rabies cases over the 10-year study period. We hypothesized that high incidence areas might change due to the increasing provision of PEP. We conducted a retrospective chart review of all patients admitted and clinically diagnosed with rabies at SLH between 2006 and 2015.

## Methods

### Ethics statement

Ethical approval was obtained from the Research and Ethical Review Board of San Lazaro Hospital, the Philippines (SLH-RERU-29022016), and the Institutional Review Board of the Institute of Tropical Medicine, Nagasaki University, Japan (No. 160303152). Both review boards approved that consent was not necessary for this retrospective study. All patient identifiers were removed from the electronic database.

### Study design and site

This study was a retrospective hospital-based case record review of all patients admitted to SLH with a clinical diagnosis of rabies between January 1, 2006, and December 31, 2015.

### Data collection

**Fig 1:**
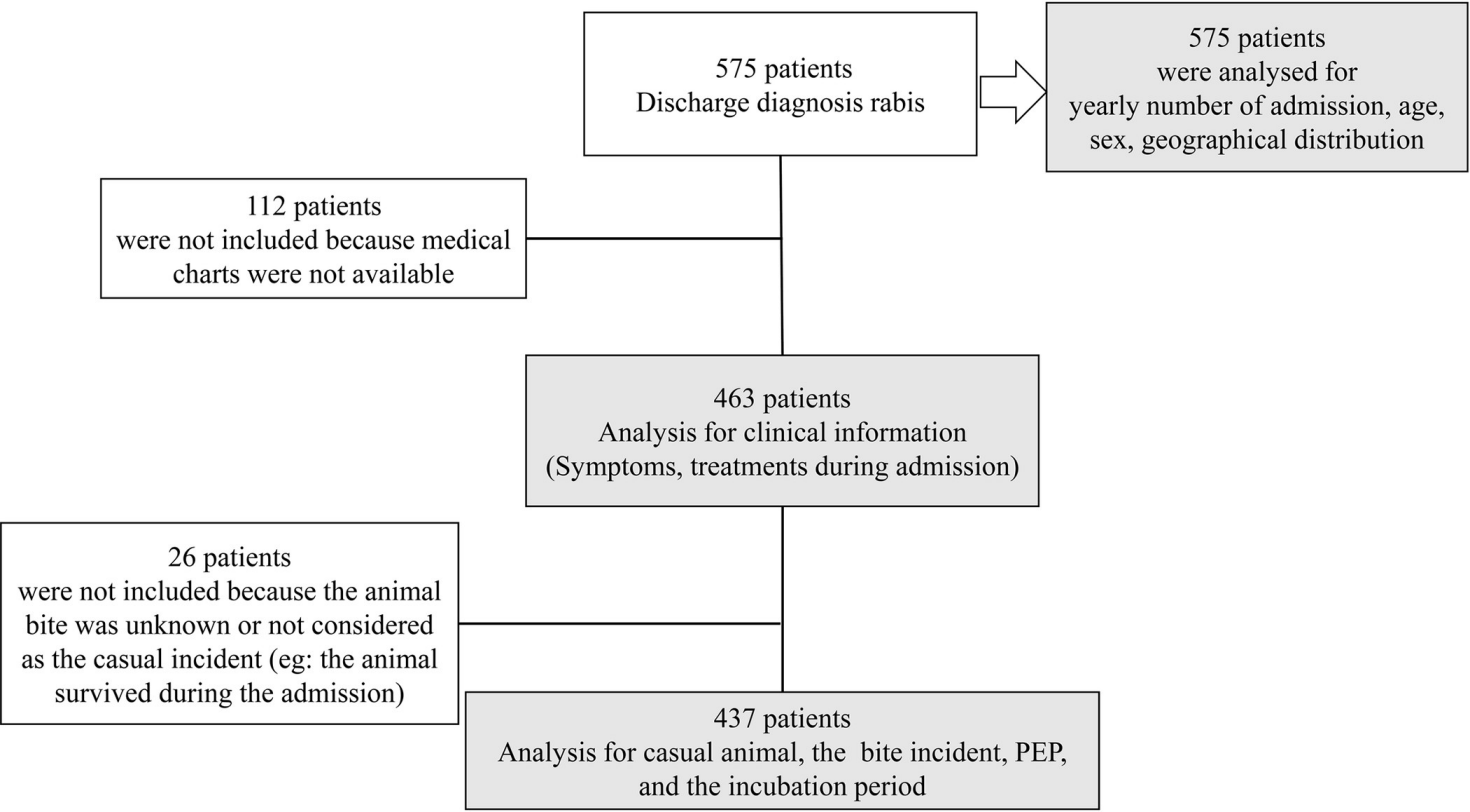
Flow chart of the inclusion for each analysis in this study.

At SLH, physicians diagnose human rabies clinically based on a history of animal bite or non-bite exposure with hydrophobia and/or aerophobia, or other sudden onset of neurological symptoms. When a clear clinical diagnosis of rabies is made, no further serological, virology, or other laboratory tests are performed for laboratory confirmation. A final diagnosis of rabies is made if the patient dies within several days of admission. Other diagnoses are made if the patient survives, such as acute central nervous system infection, psychiatric disease, Guillain-Barré syndrome, or hysteria (called rabies hysteria). From the hospital electronic database we obtained the list of all patients with a final diagnosis of rabies at SLH during the study period. The data included date of discharge, age, sex, and residential location. The flow of the analysis is shown in Fig 1. First, we analyzed the yearly admissions, and distributions, and distribution of age, sex, and residence. Next, we retrieved case medical charts from the hospital record departments and conducted chart reviews to record the medical history, information about PEP, symptoms, physical findings, vital signs on admission, reason for diagnosis, treatment, outcome, and duration of the clinical course. Finally, we obtained information on the causal animal, the bite incident, and the incubation period, excluding cases in which the animal bite incident was unknown or the biting animal survived during admission. We additionally identified rabies cases in which rabies vaccines were administered one or more times after the bite incident and noted the vaccine types and regimens. As the precise date of symptom onset is rarely available in the medical charts, we used the date of admission and date or month of exposure to calculate the incubation period. Normally, patients are admitted to this hospital 2−4 days after symptom onset if rabies is suspected [14]. As it was difficult to determine the precise incubation period, we divided the reported incubation period into periods of < 30 days, 30–90 days, 91–365 days, and >365 days.

### Statistical analysis

Data were managed using Microsoft Access (Microsoft, Redmond, WA, USA), and statistical analyses were performed using Stata software version 17.0 (StataCorp, College Station, TX, USA). We calculated the incidence rates within the NCR, Region III, and Region IV-A in 2007, 2010, and 2015, from which most rabies cases are expected to be transferred to SLH. We obtained population census data and basic maps from the Philippines Statistics Authority, United Nations Office for the Coordination of Human Affairs (OCHA) and the United States Geological Survey (USGS). We converted the home locations of patients with rabies to Global Positioning System (GPS) locations. We used Geographic Information System (GIS) software (ArcGIS version 10.5; ESRI, CA, USA) for the case mapping. Case maps were created using the residential location data. To generate heatmaps showing the density of human rabies cases during 2006–2015, we used the planar kernel density analysis tool in ArcGIS. We used the default settings of the tool and did not specify the population field or search radius. We created and overlaid two heatmaps for the periods 2006–2010 and 2011–2015 to compare the case densities between the two observation periods.

## Results

During the 10-year study period, 575 patients had a recorded diagnosis of rabies. Among them, 112 (19.5%) patients were excluded from further analysis owing to missing or lost medical charts (Fig 1 and S1 Table). An additional 26 (4.5%) patients were excluded because the animal bite incidents were unknown or were not considered the casual incidents (Fig 1).

**Fig 2:**
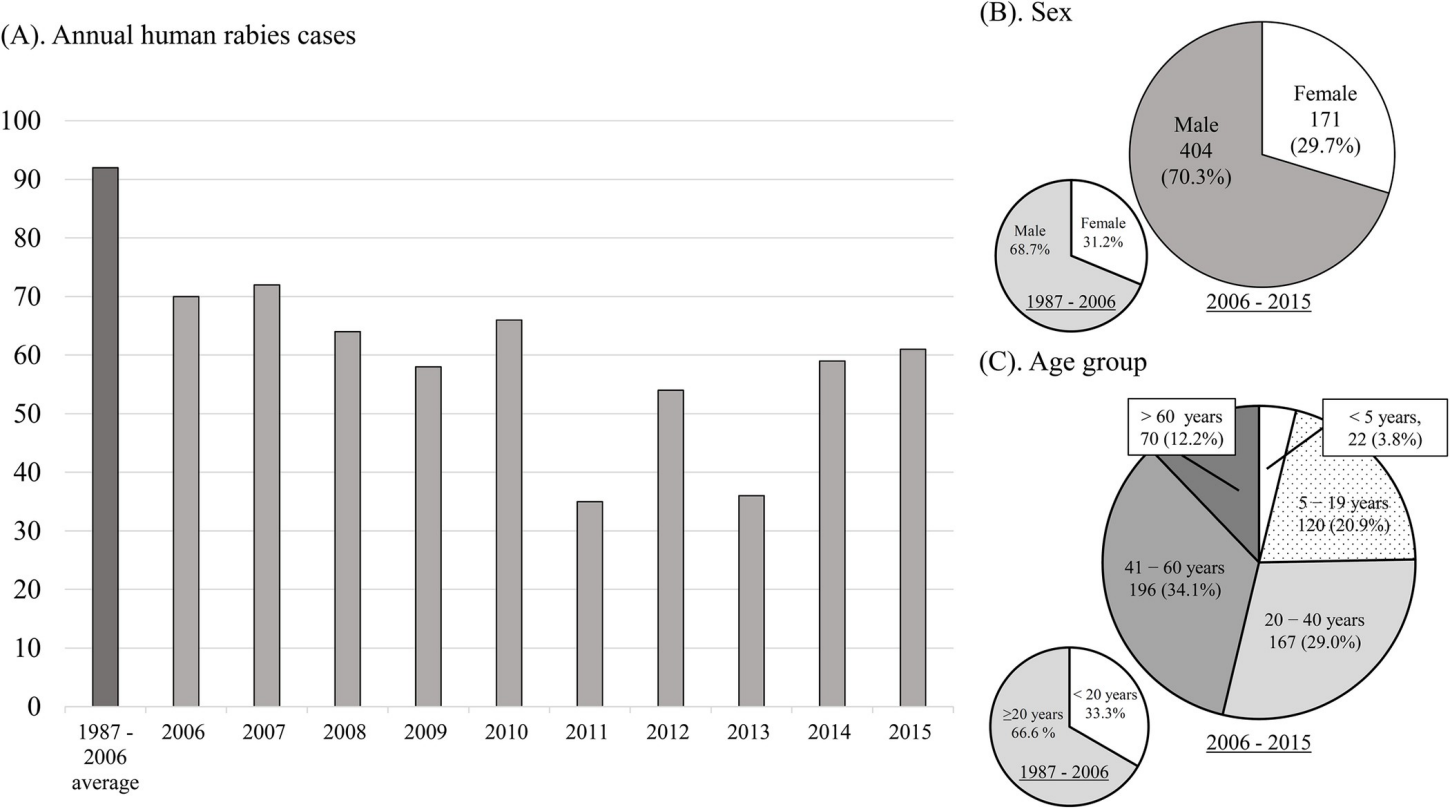
Comparisons of human rabies cases in San Lazaro Hospital between 2006–2015 and 1987–2006. (A) Annual numbers of admissions. (B) Annual numbers of patients according to sex. (C) Annual numbers of patients per age group of.

Between 2006 and 2015, the average number of yearly admissions for human rabies were lower (57.5 cases/year, range 35−72) than those reported between 1987 and 2006 (92 cases/year, range 57−119) (Fig 2A) [14]. We also observed no notable reduction in case numbers between 2006 and 2015 (Fig 2A). Most patients were male (n = 171, 70.3%), similar to previous reports (Fig 2B). The median age was 39 years (range 2−87) with 22 (3.8%) patients <5 years, 120 (20.9%) aged 5−19 years, and 433 (75.3%) aged ≥ 20 years (Fig 2C). The proportion of patients aged < 20 years was lower than that previously reported (33.3%) (Fig 2C).

**Fig 3:**
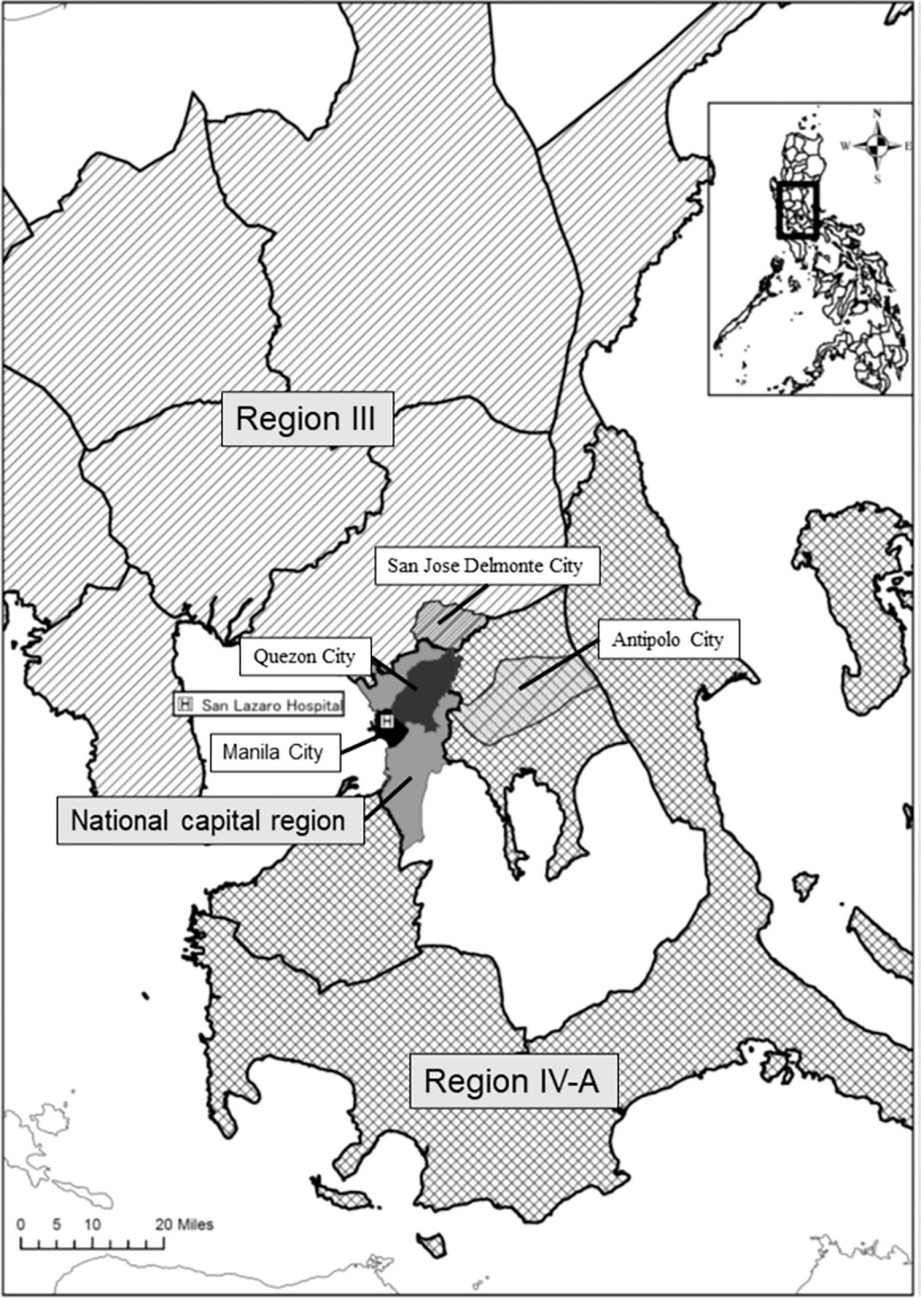
Case locations. Locations of San Lazaro Hospital, National Capital Region (gray), Manila City (black), Quezon City (dark gray), Region III (Shaded area), San Jose Delmonte City (light gray), Region IV-A (grid), and Antipolo City (light gray). Regional, provincial, city, and municipal boundary data and base maps were obtained from the United Nations Office for the Coordination of Human Affairs (OCHA). (https://data.humdata.org/dataset/philippines-administrative-levels-0-to-3).

**Fig 4:**
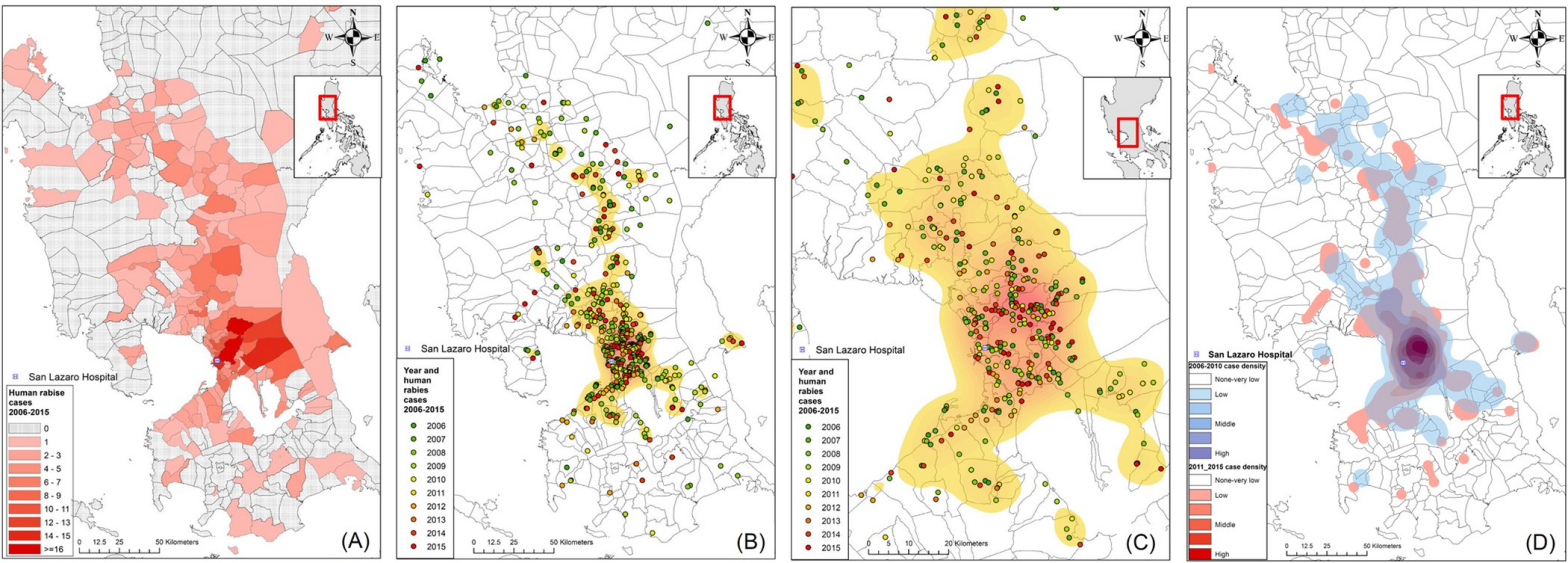
Geographical distributions of patient residential addresses among those admitted to San Lazaro Hospital with a final diagnosis of rabies between 2006 and 2015. (A) Numbers of rabies cases in municipalities or cities. (B) Case mapping and heatmaps of rabies cases in Metro Manila, Region III, and Region IV-A. (C) Enlarged scale map focusing on cases and the heatmap in Metro Manila. (D) Heatmaps showing the case densities of human rabies cases during two observational periods (2006–2010 [transparent blue] and 2011–2015 [red]); each dot represents the residential address of a rabies case, with different colors representing the year of admission. Regional, provincial, city, and municipal boundary data and base maps were obtained from the United Nations Office for the Coordination of Human Affairs (OCHA). (https://data.humdata.org/dataset/philippines-administrative-levels-0-to-3).

Most patients lived in the NCR (n = 160, 28.0%) and the adjacent Regions III (n = 197, 34.4%) and IV-A (n = 168; 29.4%) (Fig 3). The incidence rates of human rabies per 100,000 population in 2007, 2010, and 2015 were 0.1305, 0.1356, and 0.1708 in the NCR; 0.2890, 0.2965, and 0.1961 in Region III; and 0.1449, 0.1272, and 0.1041 in Region IV-A, respectively. During the study period, the city or municipality with the highest number of cases was Quezon City (43, 8.2%), followed by San Jose Delmonte City (16, 3.0%), Antipolo City (15, 2.8%), and Manila City (14, 2.7%) (Fig 3). Case mapping showed that although many cases were observed near the hospital, the cases were still widely distributed (Fig 4A–4C). Many cases were found in areas with high population density and urbanization (S2 Fig). We observed a mixture of old (green and yellow) and recent (red) cases in the high case density area identified by heatmaps (Figs 4A–4C and S2). Similar case densities were observed between 2006–2010 and 2011–2015 (Fig 4D).

Of the 463 rabies cases with available medical charts, all were diagnosed with furious rabies without laboratory confirmation. Prodromal symptoms including pain, itching, or numbness at the bite site (n = 82, 17.7%), fever ≥37.0°C (n = 224, 48.4%), and nausea/vomiting (n = 103, 22.3%) were reported more commonly compared to patients in the previous report (Table 1). The acute neurological symptoms included restlessness (n = 306, 66.1%), behavioral changes (n = 106, 22.9%), and confusion or agitation (n = 176, 38.0%). Most patients in our study showed typical symptoms of human rabies, including hydrophobia (n = 444, 95.5%) and aerophobia (n = 432, 93.3%). Other clinical symptoms included difficulty breathing (n = 190, 41.0%), hypersalivation (n = 116, 25.1%), and photophobia (n = 44, 9.5%). The patients were treated with diphenhydramine (n = 397, 85.8%), haloperidol (n = 370, 79.9%), and diazepam (n = 193, 41.7%). Several patients received intravenous fluids (n = 36, 7.8%). Only one patient (0.2%) was managed with mechanical ventilation. All patients died, most within 48 h of admission (n = 428, 92.5%). Only 2.4% (n = 11) of patients were admitted for >72 h (maximum 163 h).

**Table 1:** Clinical symptoms, interventions, and hospitalization duration among 463 patients with available medical charts and comparison to those reported previously.

Clinical symptoms		2006–2011 n = 463 N (%: 95%CI)	1987–2006 [14] N = 1839 N (%)
Prodromal symptoms	Bite site symptoms^a^	82 (17.7: 14.3–21.5)	91 (4.9)
	Fever ≥37.0°C	224 (48.4: 43.7–53.0)	173 (9.4)
	Nausea/vomiting	103 (22.3: 18.5–26.3)	119 (6.5)
Acute neurological symptoms	Restlessness	306 (66.1: 61.6–70.4)	169 (9.2)
	Confusion/agitation	176 (38.0: 33.6–42.6)	
	Behavioral change	106 (22.9: 19.1–27.0)	
	Seizure	6 (1.3: 0.5–2.8)	
	Paralysis	2 (0.4: 0.1–1.6)	71 (3.9)
Autonomic dysfunction	Hydrophobia	444 (95.9: 93.7–97.5)	1839 (100) ^b^
	Aerophobia	432 (93.3: 90.6–95.4)	1756 (95.5)
	Difficulty breathing	190 (41.0: 36.5–45.7)	
	Dyphagia	119 (25.7: 21.8–29.9)	
	Hypersalivation	116 (25.1:21.2–29.3)	124 (6.7)
	Photophobia	44 (9.5: 7.0–12.5)	23 (1.3)
Intervention	Diphenhydramine	397 (85.8: 82.2–88.8)	
	Haloperidol	370 (79.9: 76.0–83.5)	
	Diazepam	193 (41.7: 37.2–46.3)	
	IV fluid	36 (7.8: 5.5–10.6)	
	Mechanical ventilator	1 (0.2: 0.00–1.2)	
Hospitalization duration (hours)	≤24 hours	324 (70.0: 65.6–74.1)	
	25–48 hours	104 (22.5: 18.7–26.5)	
	49–72 hours	23 (5.0: 3.2–7.4)	
	73–168 hours	11 (2.4: 1.2–4.2)	
	Unknown/no record	1 (0.2: 0.00–1.2)	

95%CI: 95% confidence interval. ^a^pain, itching, or numbness; IV: intravenous ^b^clinical rabies was defined as the presence of hydrophobia in this study.

Analysis of the medical charts of the 437 patients with an animal exposure history showed that most possible causal animals were dogs (n = 421, 96.3%) and cats (n = 16, 3.7%), similar to a previous report (Table 2). Of the 437 animals, 113 (25.9%) and 78 (17.9%) were pets and stray animals, respectively, which were lower than those reported previously [14]. The exposure type was mostly animal bite (n = 415, 95.0%). In most cases, a single bite rather than multiple bites was documented (n = 310, 74.7% vs. n = 13, 3.1%, respectively). The most common bite locations were the lower extremities (34.6%), followed by the upper extremities (26.3%); fingers (10.1%); and face, head, or neck (4.8%). The incubation period was ≤30 days in 99 (22.7%) patients and 30–90 days in 184 (42.1%) patients. Approximately 10% of the patients (41 cases) had an incubation period of >1 year (maximum 10 years).

**Table 2:** Characteristics of the casual animals, exposures, and post-exposure prophylaxis among 437 patients reporting animal exposures compared to a previous study.

		2006–2011 n = 467 N (%: 95%CI)	1987–2006 [14] N = 1839 N (%)
Animal type	Dog	421 (96.3: 94.1–97.9)	1639 (97.1)
	Cat	16 (3.7: 2.1–5.9)	49 (2.9)
Pet or stray	Pet	113 (25.9: 21.8–30.2)	581 (35.5)
	Stray	78 (17.8: 14.3–21.8)	1057 (64.5)
	Unknown/not recorded	246 (56.3: 51.5–61.0)	
Animal condition	Died	88 (20.1: 16.5–24.2)	
	Euthanasia	100 (22.9: 19.0–27.1)	
	Unknown/not recorded	249 (57.0: 52.2–61.7)	
Exposure contact	Bite	415 (95.0: 92.5–96.8)	1814 (98.6)
	Scratch	10 (2.3: 1.1–4.2)	
	Lick	7 (1.6: 0.6–3.3)	
	Other^a^	5 (1.1: 0.4–2.6)	21 (84)
Bite exposure (n = 415)	Single	310 (74.7: 70.2–78.8)	
	Multiple	13 (3.1: 1.7–5.3)	
	Unknown/not recorded	92 (22.2: 18.3–26.5)	
Body sites of bite exposure(s)^b^	Face, head, or neck	21 (4.8: 3.0–7.3)	
	Fingers	44 (10.1: 7.4–13.3)	
	Upper extremities	115 (26.3: 22.2–30.7)	
	Lower extremities	151 (34.6: 30.1–39.2)	
Incubation period (days)	≤30	99 (22.7: 18.8–26.9)	292 (16.0)
	30–90	184 (42.1: 37.4–46.9)	498 (27.3)
	91–365	81 (18.5: 15.0–22.5)	785 (43.0)
	>365	41 (9.4: 6.8–12.5)	251 (13.7)
	Unknown/no record	33 (7.6: 5.3–10.4)	
Rabies vaccine and RIG as post-exposure prophylaxis	No vaccine and No RIG	395 (90.4: 87.2–93.0)	1808 (98.3)
	1 dose, no RIG	20 (4.6: 2.8–7.0)	
	1 dose + RIG	4 (0.9: 0.2–2.3)	8 (0.4)
	2 doses No RIG	7 (1.6: 0.6–3.3)	22 (1.1)^c^
	2 doses + RIG	1 (0.2: 0.00–1.3)	
	≥3 doses, No RIG	8 (1.8: 0.8–3.6)	
	≥3 doses + RIG	2 (0.5: 0.0–1.6)	1 (0.05)^e^

95%CI: 95% confidence interval. RIG, rabies immunoglobulin.

^a^Most other reported exposures were dog meat consumption or cooking raw animal meat

^b^multiple choice

^c^the doses of rabies vaccines were not clearly indicated

^e^RIG treatment was delayed for 2 days after animal exposure. The patient experienced multiple facial bites. RIG was administered intramuscularly.

Among 437 patients with animal exposure history, 395 (90.4%) did not receive a rabies vaccine or RIG, while 42 (9.6%) received one or more rabies vaccines as PEP. Ten patients (2.3%) received >3 doses of a rabies vaccine, as recommended by the WHO, but only two patients (0.5%) received RIG (Table 3). One was a 5-year-old boy who was bitten on his face by a dog. He was administered a purified chick embryo cell vaccine (PCECV) on days 0, 3, and 7, and RIG on day 0, starting on the same day as the animal bite, although the vaccination dose was not recorded. Despite receiving PEP, the patient died 21 days after the dog bite. The second patient was a 2-year-old girl with multiple bites on her face, neck, and upper extremities. She was administered 0.1 mL intradermal PCECV as PEP on days 0, 2, 7, and 21, and RIG on day 0, starting the day after the dog bite. She died 30 days after exposure to the animal. None of the 437 patients received PEP before exposure.

**Table 3:** Characteristics of patients with rabies who received >3 doses of post-exposure prophylaxis.

Age (years)	Month and year of admission	Single or multiple bites	Exposure site	Vaccine doses	RIG	Doses administered according to schedule	Duration between bite and admission
36	Feb 2007	−	−	4 doses	−	−	2 months
64	Jan 2010	Single	Legs	5 doses	−	−	1 year
48	Jan 2012	Single	Arms	4 doses	N	−	2 months
3	May 2012	Single	Legs	4 doses	−	−	30 days
20	Sep 2013	−	−	4 doses	−	−	6 months
2^a^	Jan 2014	Multiple	Face, head, arm	4 doses	Y	Y	30 days
5^a^	Feb 2014	Single	Face	3 doses	Y	Y	21 days
25	April 2015	−	−	4 doses	−	−	60 days
56	Jun 2015	Single	Face	3 doses	N	N	30 days
14	Dec 2015	−	−	4 doses	−	−	30 days

RIG: rabies immunoglobulin; −: no record or unknown; N: no; Y: yes.

^a^patients received the complete post-exposure prophylaxis regime (RIG + series of vaccinations).

## Discussion

During the 10-year study period, human rabies cases were continuously admitted to the referral center in Metro Manila and virus transmission from infected animal bites persisted in the surrounding regions. Case maps and heatmaps demonstrated that rabies occurred continuously in similar areas during the 10-year study period. Most admitted rabies patients were adult men who did not seek PEP. The characteristics of human patients with rabies were similar to those described in this hospital between 1987 and 2006. We identified two cases of possible PEP failure.

Our results reflect the status of rabies cases around the NCR in the Philippines. Most human rabies cases in the NCR, Region III, and Region IV-A were referred to our hospital. An average of 57.5 cases annually were documented in this study, 23.1% of the yearly average of 248.7 cases in the Philippines [11]. The incidence rate per 100,000 population varied between 0.1041 and 0.2965 in these regions and was similar to that reported in China between 1996 and 2007 [15]. The characteristics of the patients with rabies reported previously in this hospital between 1978 and 2006 were broadly similar to those of the current study; however, the average number of cases each year (92 cases) was higher than that in our study (57.5 cases) [14]. The case maps and heatmaps showed that human rabies occurred continuously in similar areas during the 10-year study period. Studies in China and Brazil have shown decreasing trends and changing distributions of human rabies cases owing to successful rabies control programs [16–18]. Our findings are contrary to these findings, likely because the control programs, particularly mass animal vaccination, have been limited to certain areas of the Philippines. The National Program reported vaccine coverage rates in the NCR, Region III, and Region IV-A of 32.3%, 49.9%, and 38.9%, respectively, in 2015 [10]. Several recent community studies have shown that the dog population is much higher than the figures calculated by the recommendation of the rabies control program based on a 1:10 dog-human ratio [19,20]. Therefore, the dog vaccination coverage in many regions may be overestimated and far below the target of 70%. A careful analysis of rabies vaccine coverage in domestic dogs and strengthening of control programs is needed in areas where human rabies cases continue to be reported. A careful analysis of rabies vaccine coverage in domestic dogs and a strengthening of control programs is needed in areas where human rabies cases continue to be reported. The elimination of rabies in dogs through mass vaccination is cost-effective and has been successfully achieved in many areas [21–23].

All cases in this study were the furious rabies type and no patients were diagnosed with paralytic rabies. Paralytic rabies has rarely been reported in the Philippines [14]. This contrasts with reports from other countries, where up to one-third of human rabies cases can be paralytic rabies [24,25]. A study in Indonesia showed that 22% of human rabies cases were paralytic rabies [26], whereas reports from China and the Democratic Republic of Congo showed similar findings to our study [27,28]. Paralytic rabies may be misdiagnosed in the Philippines, or circulating strains in the Philippines may differ and cause fewer paralytic cases [29,30]. Further investigations are needed to understand the importance of paralytic rabies virus in this area. In the present study, the diagnosis of rabies was based on the clinical history, symptoms, and signs. The presence of hydrophobia and/or aerophobia is a key component when patients with suspected rabies are referred to this hospital.

The incubation periods observed in this study were comparable to those reported previously at this hospital [14,17,28,31]. Long incubation periods have been reported in some reports [14,31,32]. Incubation periods of >1 year were observed in 41 cases, with the longest incubation period of 10 years in our study. The accuracy of these data was limited due to the retrospective nature of the analysis, and we were not able to perform further detailed investigations on these cases. In this study, most of the patients died within 20–30 hours from the time of admission and may have presented to the hospital relatively late in the progression of symptoms.

Most rabies patients in this study were adult men, similar to a previous study conducted in this hospital [14]. A study analyzing the characteristics of individuals attending animal bite treatment centers in the Philippines reported more children or young adults seeking PEP compared to middle-aged adults [11]. Although animal bite exposures among adults might be lower than those of children, these findings indicated that middle-aged men may be less likely to seek medical care after an animal bite compared to younger age groups [11,33,34]. Strengthening education campaigns targeting older men should be considered to increase their likelihood of seeking medical treatment.

Most rabies patients did not receive a rabies vaccine or RIG, although more patients (n = 42; 9.6%) received at least one vaccine compared to the previous study in this hospital (n = 31; 1.7%) [14]. Among the 42 cases administered the vaccine, 20 (47.6%) were administered one dose, possibly because of a lack of time or financial considerations, as reported elsewhere [34]. Only 10 patients (2.3%) received ≥3 doses of rabies vaccines, in accordance with the WHO recommended regimen. We were unable to determine the routes of vaccine administration and manufacture. ID regimes were adopted by the national guideline in 1997 [8,9]. SLH started the ID regime in 1996; thus, it is likely that most health centers started it around the same year. Therefore, the patients in our study who received a rabies vaccine as PEP were likely to receive the vaccine via the ID route. It remains common to consult a traditional healer after an animal bite in the Philippines [34]. We were unable to determine the proportion of patients with rabies attending traditional healers or the common reasons for not receiving PEP. Further investigation is needed to clarify the health-seeking behavior of patients with rabies.

We identified two patients who died of rabies despite receiving complete PEP (Table 3). Both patients were young, (5 and 2 years of age, respectively), experienced short incubation periods (20–28 days), and were bitten on the face. The method of RIG administration and manufacture of the vaccine were not identified in these cases. Treatment failure mostly occurs due to inappropriate wound washing, delayed treatment, or non-completion of PEP. PEP failure in patients with full course is rare. Guo *et al* reported 31 patients who had completed PEP but died of rabies among 10,971 human rabies cases in China [28]. Another study from China reported 19 cases of PEP failure among 711 human rabies cases [35]. Ren *et al* observed one case of PEP failure among 201 human rabies cases: a 5-year-old boy who was seriously bitten on his face/head by a stray dog [36]. A study in Cambodia reviewed 1,739 bite victims bitten by rabid dogs and reported three cases of possible PEP failure (0.17%; 95% CI: 0.03–0.50) [37]. Wilde *et al* described eight rabies cases with PEP failure [38]. Several case reports have also described single or multiple cases of PEP failure [39–41]. Many of these PEP failures occurred in young children and individuals with head/face bites. The virus incubation period following such injuries is often short (≤30 days). RIG infiltration is often difficult in small children with head/face injuries, which can lead to insufficient treatment. Direct inoculation of the virus into peripheral nerves might also cause PEP failure. Because rabies is almost universally fatal, more treatment options are needed for high-risk bite victims, such as individuals with face or head bite injuries, small children, or individuals bitten by laboratory-confirmed rabid animals. RIG shortages often occur in endemic areas because of the increasing demands due to animal bites. During such shortages, RIG prioritization is necessary. To identify high-risk bite victims, a higher WHO exposure category (Cat IV) should be considered. This category might be useful when RIG supply is limited and prioritization is necessary. Furthermore, add-on or alternative treatments for RIG should be assessed in this group. These high-risk bite victims should be carefully observed, and longer follow-up is necessary after PEP. A recent study demonstrated that favipiravir (T-705) is active against rabies virus in mice and may be a potential alternative or add-on treatment to RIG [42,43].

Our study had some limitations. This report is from a single health center study and did not cover all human rabies cases in the Philippines. Although our study might fail to determine the true incidence rate and some hot spots, this hospital does treat most human rabies cases in the NCR, Regions III and IV, and the referral system of human rabies cases did not change during the study period. Our analysis clarified the changing patterns and distribution of human rabies cases during the study period. A further limitation was that our case mapping showed the living places (home addresses) of the patient but not the locations where the rabid animals were encountered. None of the cases in our study were laboratory-confirmed because of a lack of diagnostic capacity for SLH. The retrospective design of the study meant that the data relied on the medical charts written by the attending physicians and questions from the physicians to patients or relatives. Our data on animal exposure and PEP treatments may be affected by recall bias, and we were unable to describe the WHO exposure categories, administration method of RIG, reasons for not seeking PEP, and details about the casual animals. Although one community survey in the Philippines revealed common reasons for not accessing medical treatment among individuals bitten by animals [11,34], the reasons among human rabies patients have not been studied. Prospective studies with laboratory confirmation are needed to clarify these issues, including the factors associated with not receiving PEP and the possible cases of PEP failure.

## Conclusion

The results of our study showed that human rabies patients were continuously admitted to the hospital between 2006 and 2015, with no notable decline over the study period. The clinical characteristics were largely similar to those of the patients admitted to this hospital between 1987 and 2006. The geographical areas in which human rabies cases commonly occurred also did not change. Few patients received PEP and there were two suspected cases of PEP failure. The retrospective design of this study was a limitation; thus, prospective studies are required.

## Supporting information

S1 Table Characteristics of human patients with rabies who were excluded from further analysis due to missing or lost medical charts (n = 112). (PDF)

S1 Fig The numbers of human rabies cases and animal bite victims attending animal bite treatment centers in the Philippines between 2007 and 2015. These data were obtained from the National Rabies Prevention and Control Program in the Philippines. Manual of Procedures (2019). https://doh.gov.ph/sites/default/files/publications/Rabies%20Manual_MOP_2019%20nov28.pdf
(TIF)

S2 Fig Geographical distributions of the residential addresses of patients admitted to San Lazaro Hospital with a final diagnosis of rabies between 2006 and 2015 on geological, population, and population density base maps. (A) Case and geological maps. The base maps were obtained from the U.S. Geological Survey (USGS) and are in the public domain. https://earthexplorer.usgs.gov/scene/metadata/full/5e83d0b656b77cf3/LC81160502016044LGN01/ (B) Case and population maps per city/municipality in Metro Manila and Regions III and IV-A. (C) Case mapping and population maps per city/municipality in Metro Manila (enlarged map in B). (D) Case and population density maps per city/municipality per square kilometer in Metro Manila and Regions III and IV-A. (E) Case mapping and population density maps per city/municipality per square kilometer in Metro Manila (enlarged map of D). Each dot represents the residential address of rabies cases, with different colors representing the years of admission between 2006 and 2015. Regional, provincial, city, and municipal boundary data and base maps were obtained from the United Nations Office for the Coordination of Human Affairs (OCHA). (https://data.humdata.org/dataset/philippines-administrative-levels-0-to-3).
(TIF)

S1 Dataset Study data. (XLS)
