# Supplementary material for: Clinical, epidemiological, and spatial features of human rabies cases in Metro Manila, the Philippines from 2006 to 2015
Source: PLoS Negl Trop Dis. 2022 Jul 19;16(7):e0010595. doi: 10.1371/journal.pntd.0010595 (PMC9295989; doi:10.1371/journal.pntd.0010595)
Supplement: S1 Table — (PDF) [file pntd.0010595.s001.pdf]

S1 Table

Characteristics of human rabies patients who were excluded from further analysis because of missing or lost medical charts (n=112)

		N	%
Year	2006	45	40.2%
	2007	14	12.5%
	2008	12	10.7%
	2009	16	14.3%
	2010	0	0.0%
	2011	4	3.6%
	2012	6	5.4%
	2013	5	4.5%
	2014	4	3.6%
	2015	6	5.4%
Age group	<5year	5	4.5%
	5-18 years	21	18.8%
	19-40years	26	23.2%
	41-60years	45	40.2%
	>60 years	15	13.4%
Sex	Male	36	32.1%
	Female	76	67.9%
Region	NCR	27	24.1%
	Region 1	6	5.4%
	Region 2	2	1.8%
	Region 3	35	31.3%
	Region 4	38	33.9%
	Region 5	1	0.9%
	Unknown	3	2.7%
