# Supplementary figures and images for: Clinical, epidemiological, and spatial features of human rabies cases in Metro Manila, the Philippines from 2006 to 2015
Source: PLoS Negl Trop Dis. 2022 Jul 19;16(7):e0010595. doi: 10.1371/journal.pntd.0010595 (PMC9295989; doi:10.1371/journal.pntd.0010595)

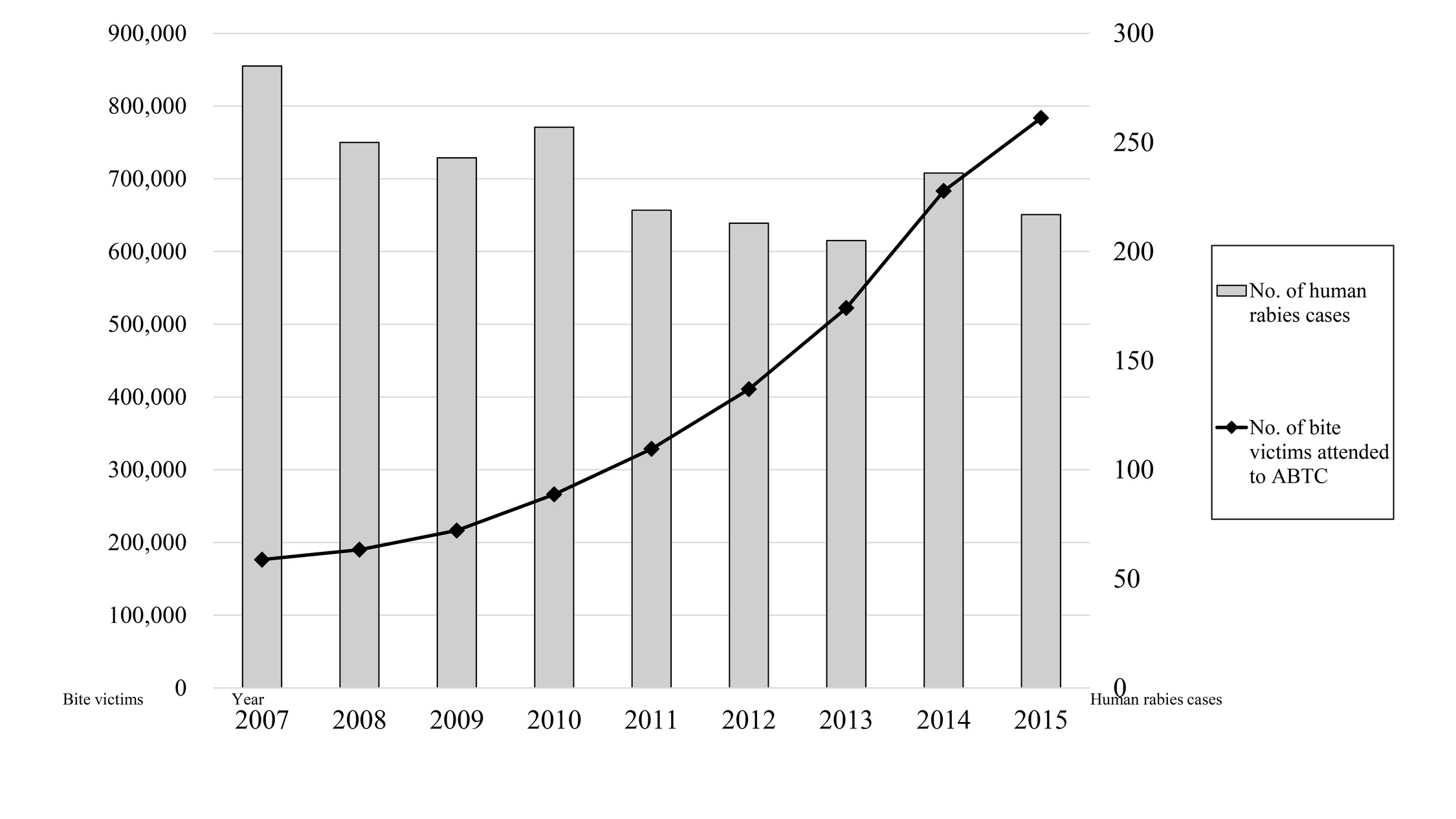

Supplement: S1 Fig — These data were obtained from the National Rabies Prevention and Control Program in the Philippines. Manual of Procedures (2019). https://doh.gov.ph/sites/default/files/publications/Rabies%20Manual_MOP_2019%20nov28.pdf (TIF) [file pntd.0010595.s002.tif]

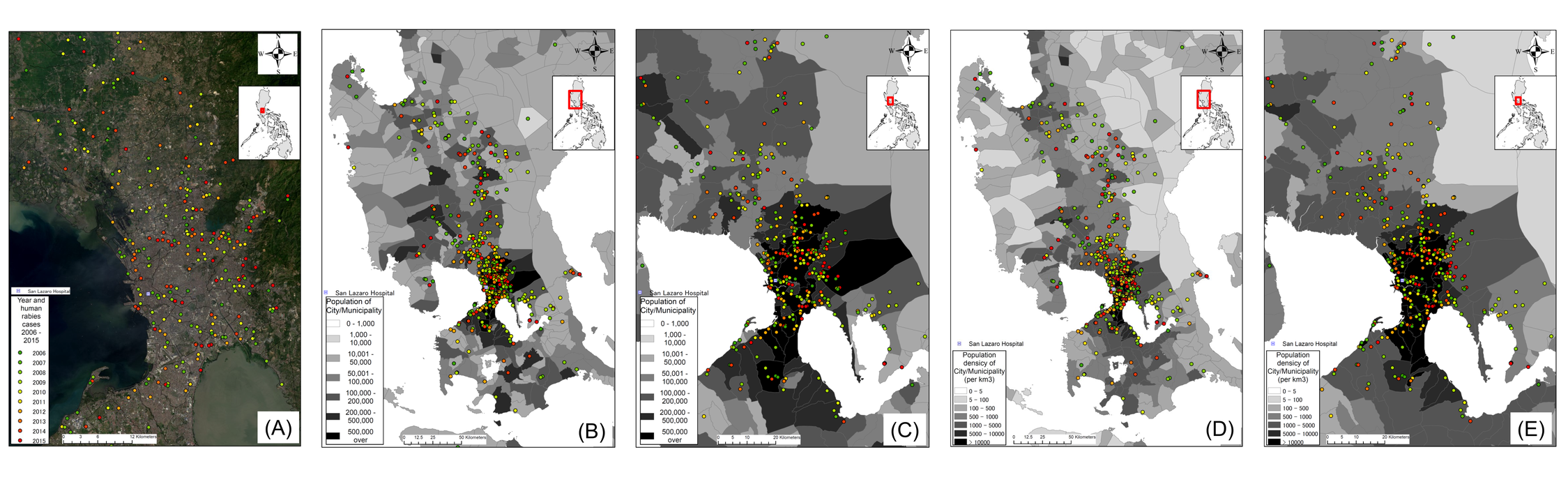

Supplement: S2 Fig — (A) Case and geological maps. The base maps were obtained from the U.S. Geological Survey (USGS) and are in the public domain. https://earthexplorer.usgs.gov/scene/metadata/full/5e83d0b656b77cf3/LC81160502016044LGN01/ (B) Case and population maps per city/municipality in Metro Manila and Regions III and IV-A. (C) Case mapping and population maps per city/municipality in Metro Manila (enlarged map in B). (D) Case and population density maps per city/municipality per square kilometer in Metro Manila and Regions III and IV-A. (E) Case mapping and population density maps per city/municipality per square kilometer in Metro Manila (enlarged map of D). Each dot represents the residential address of rabies cases, with different colors representing the years of admission between 2006 and 2015. Regional, provincial, city, and municipal boundary data and base maps were obtained from the United Nations Office for the Coordination of Human Affairs (OCHA). (https://data.humdata.org/dataset/philippines-administrative-levels-0-to-3). (TIF) [file pntd.0010595.s003.tif]
